# Outcome of Allogeneic Penetrating Limbo-Keratoplasty: A Single-Center Retrospective Cohort Study

**DOI:** 10.3390/jcm14248958

**Published:** 2025-12-18

**Authors:** Marie Ella Horstmann, Alexander K. Schuster, Norbert Pfeiffer, Joanna Wasielica-Poslednik

**Affiliations:** Department of Ophthalmology, University Medical Center of the Johannes Gutenberg-University Mainz, 55131 Mainz, Germanyjoanna.wasielica-poslednik@unimedizin-mainz.de (J.W.-P.)

**Keywords:** limbo-keratoplasty (Limbo-PK), limbal stem cell deficiency (LSCD), corneal graft, corneal transplantation, perforating keratoplasty (PK)

## Abstract

**Introduction:** Allogeneic penetrating limbo-keratoplasty (limbo-PK) is one of the surgical methods for the treatment of limbal stem cell deficiency (LSCD). We report real-life results on different entities. **Methods**: Patients treated with limbo-PK at the Department of Ophthalmology of the University Medical Center Mainz were evaluated retrospectively. The primary endpoint was the epithelialization of the graft one year postoperatively. In addition, the postoperative best corrected visual acuity (BCVA), ocular concomitant diseases, drug treatment, and the need for further eye surgery postoperatively were examined. **Results**: We included 14 eyes of 13 patients (4 female) aged 59.8 ± 14.1 years who underwent limbo-PK between 2020 and 2024. Indications for limbo-PK included chemical burns (*n* = 4), blast injuries (*n* = 4), thermal burns (*n* = 2), trauma (*n* = 1) graft-versus-host disease (*n* = 1), and ectrodactyly-ectodermal dysplasia (EEC) (*n* = 1). The mean preoperative BCVA was 2.2 ± 0.6 logMAR (range: light perception to 0.7 logMAR). Four limbo-PK-grafts were HLA-typed. All limbo-PKs were combined with amniotic membrane transplantation; three with cataract surgery and one with tarsorrhaphy. Postoperatively, all patients received local immunosuppression, and 12 (85.7%) received additional systemic immunosuppression. At one-year follow-up mean BCVA increased to 1.0 ± 0.7 logMAR (range: 2.3 to 0.1, *p*-value = 0.03) and 11 of 14 eyes showed a functional graft with closed epithelium. In the further postoperative course, four patients needed a further Limbo-PK due to graft failure (*n* = 2), immune graft rejection after stopping local immunosuppressive therapy (*n* = 1) and perforation of the graft in a severe case of GvHd (*n* = 1). **Conclusions**: Limbo-PK is an effective surgical method for the treatment of LSCD. In our study cohort, we observed a significant improvement in mean BCVA one year postoperatively, with a functional, epithelialized graft achieved in 11 of 14 eyes.

## 1. Introduction

The limbus is the transitional zone between the cornea and sclera. It houses limbal stem cells (LSCs), which are responsible for the proliferation and differentiation of the corneal epithelium and are essential for wound healing. A deficiency of these cells, caused by genetic disorders, immune-mediated diseases, or trauma, leads to limbal stem cell deficiency (LSCD) [1,2]. Depending on the etiology, LSCD can be classified as primary or secondary. Primary limbal stem cell deficiency arises mainly from congenital and hereditary disorders. Recognized causes include congenital aniridia, erythrokeratodermia of the Burns type, keratitis associated with endocrine insufficiency, and ectrodactyly–ectodermal dysplasia (EEC) syndrome. In contrast, secondary limbal stem cell deficiency develops as a result of acquired damage to the limbal stem cell niche. Common etiological factors include chemical injuries caused by alkalis or acids, thermal burns, contact lens–related keratopathy, and repeated surgical interventions involving the limbus. In addition, inflammatory and immune-mediated conditions such as Stevens–Johnson syndrome and scarring pemphigoid are well-established causes. Secondary limbal stem cell deficiency may also result from infectious processes, including microbial keratitis, as well as from toxic or iatrogenic influences such as irradiation, chemotherapy, and graft-versus-host disease [3].

LSCD results in vascularization, pannus formation, persistent epithelial defects, stromal scarring, chronic inflammation, and vision loss [1]. The corneal surface appears irregular and dull, with epithelial and subepithelial opacities. In most cases, recurrent or persistent corneal erosions occur, which can lead to corneal ulcers and even perforation. The presence of goblet cells in the corneal epithelium is evidence of limbal insufficiency [4,5].

The therapy of LSCD depends on the cause, laterality, and severity of the disease. There is no topical or systemic causative treatment for LSCD [6]. Conservative therapy includes intensive lubrication, autologous serum eye drops, plasma rich in growth factors, and the use of antibiotics and immunosuppressive drugs, depending on the presence or absence of corneal epithelialization, microbial infections, and the degree of inflammation. According to the International LSCD Working Group, established by the Cornea Society in 2012, the following measures should be considered before surgical LSC restoration: “Eyelid and conjunctival reconstruction, anti-inflammatory therapy, treatment of dry eye and meibomian gland dysfunction, minimization of ocular surface toxicity from medications, topical medications that promote epithelialization, and the use of scleral lenses” [6].

A range of surgical options is available, as LSC restoration is currently only feasible through surgical intervention. Possible pathways for the treatment of limbal stem cell deficiency are recommended by the International LSCD Working Group [6]: The surgical management of limbal stem cell deficiency is guided by disease stage and laterality. In unilateral and bilateral stage I and IIA disease, treatment initially consists of observation or conservative management with medical therapy and contact lens use. In cases showing progression, surgical intervention such as superficial corneal scar excision (SSCE) or pannus removal combined with amniotic membrane transplantation (AMT) is performed.

In more advanced disease stages (IIB and III), treatment strategies differ according to laterality. In unilateral limbal stem cell deficiency, management begins with medical therapy and contact lens support, followed by autologous limbal stem cell transplantation when indicated. Additional therapeutic options may be considered depending on individual clinical circumstances. In contrast, bilateral limbal stem cell deficiency requires alternative surgical approaches, as autologous tissue is unavailable. In these cases, allogeneic limbal stem cell transplantation represents a standard option, while keratoprosthesis or cultivated oral mucosal epithelial transplantation (COMET) may be employed in selected patients. However, all these procedures have risks and limitations. Transplantation of autologous tissue has the advantage of eliminating the need for immunosuppressive therapy [6,7]. However, any intervention on the limbus carries the risk of inducing iatrogenic limbal insufficiency. This procedure requires transplantation of 40–50% of the donor limbus, which can lead to iatrogenic LSCD in the healthy eye [6,8]. Ex vivo cultivation of LSCs is limited to specialized centers, very expensive, and not widely available.

In bilateral cases of severe LSCD, allo-LSC transplantation combined with penetrating keratoplasty (limbo-PK) or deep anterior lamellar keratoplasty (limbo-DALK) or implantation of a keratoprosthesis are the only vision-restoring therapies [8,9,10]. Keratoprosthesis techniques such as the Boston Keratoprosthesis or, particularly, Osteo-Odonto-Keratoprosthesis (OOKP) are costly and not widely available. OOKP in particular is performed only in a few specialized centers.

**Figure 1:**
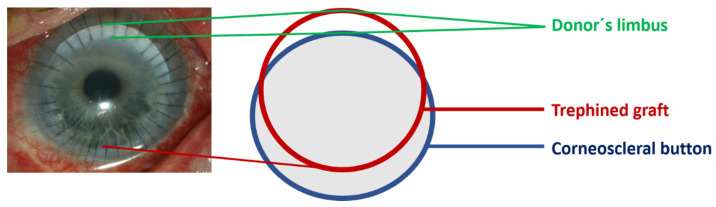
Schematic presentation of a penetrating limbo-keratoplasty. An eccentric trepanation of the donor tissue is performed to obtain a limbal donor crescent.

Limbo-PK, first introduced by Reinhard T. et al. in 1993, combines penetrating keratoplasty with transplantation of allo-LSCs from the same donor [8]. Eccentric trepanation of the corneoscleral button results in a graft that includes LSCs covering approximately 40% of the circumference (Figure 1). These grafts are then centrally fixed like conventional PK [8]. The benefits of limbo-PK have been demonstrated in patients with complete limbal stem cell failure [11]. Additionally, its indications have been expanded to include epithelial and stromal corneal dystrophies such as lattice and granular corneal dystrophy, which are associated with mutations in the transforming growth factor beta-induced (TGFBI) gene. In these cases, the aim of limbo-PK is to prevent or delay the recurrence of corneal dystrophy in the graft [11].

The advantages of limbo-PK include the rapid restoration of visual acuity and the simultaneous transplantation of limbal stem cells in a single procedure without requiring special devices such as keratoprostheses. Furthermore, it can be performed in centers that already perform conventional keratoplasties without additional costs. However, the prognosis of this technique is limited due to a higher rate of immune-mediated graft rejection and the potential failure of the transplanted limbal stem cells, leading to recurrence of LSCD [12,13].

The aim of this study is to evaluate our experience with this surgical method in a German tertiary center.

## 2. Materials and Methods

### 2.1. Study Design

This retrospective cohort study was conducted at the Department of Ophthalmology of the Johannes Gutenberg University Mainz, Germany. We analyzed data from all consecutive patients who underwent limbo-PK between 2020 and 2024. According to local law (‘Landeskrankenhausgesetz’ §36, §37), no ethical approval was required for this retrospective review. This cohort study was conducted in accordance with the ethical principles for medical research involving human participants of the Declaration of Helsinki, developed by the World Medical Association.

Limbo-PK was performed in patients with LSCD stages IIB and III who had reduced vision due to corneal opacity and who, in our clinical assessment, had no realistic chance of success with PK alone without limbal transplantation. Most of these patients had undergone previous procedures, such as SLET or PK, which had been unsuccessful in the past. We present the patients’ characteristics, including preoperative procedures, pre- and postoperative VA, and further postoperative course of all patients in Table 1.

The clinical diagnosis of LSCD was made according to anamnesis and clinical features typical of the disease. We collected the following baseline data of the recipients: age, gender, ocular history (especially regarding ocular concomitant diseases and the cause of LSCD), systemic and ocular medication, and the preoperative BCVA.

The primary endpoint was the epithelialization of the graft one year postoperatively and at the last follow-up. In addition, the postoperative best-corrected visual acuity (BCVA), ocular concomitant diseases, drug treatment, and the need for further eye surgery postoperatively were examined.

### 2.2. Donor Tissue

Donor tissue has been obtained from the Eye Bank of Rhineland-Palatinate, Mainz, Germany.

At the eye bank, the tissue has been stored at 34 °C in dextran-free cell culture medium (P04-09701 PanBiotech, Aidenbach, Germany) and transferred to 6% dextran-containing cell culture medium (P04-09702 PanBiotech, Aidenbach, Germany) at least 24 h before use. Both media were supplemented with gamma-irradiated fetal calf serum at 2% or 10% in the case of donors younger than 40 years (S-FBS-AU-035, Serana, Pessin, Germany). The diagnosis of malignant disease excludes donors for limbo-PK. The HLA-typed donor tissue was obtained from ETB-BISLIFE Multi Tissue Center (Haarlem, The Netherlands).

### 2.3. Surgery and Postoperative Care

**Figure 2:**
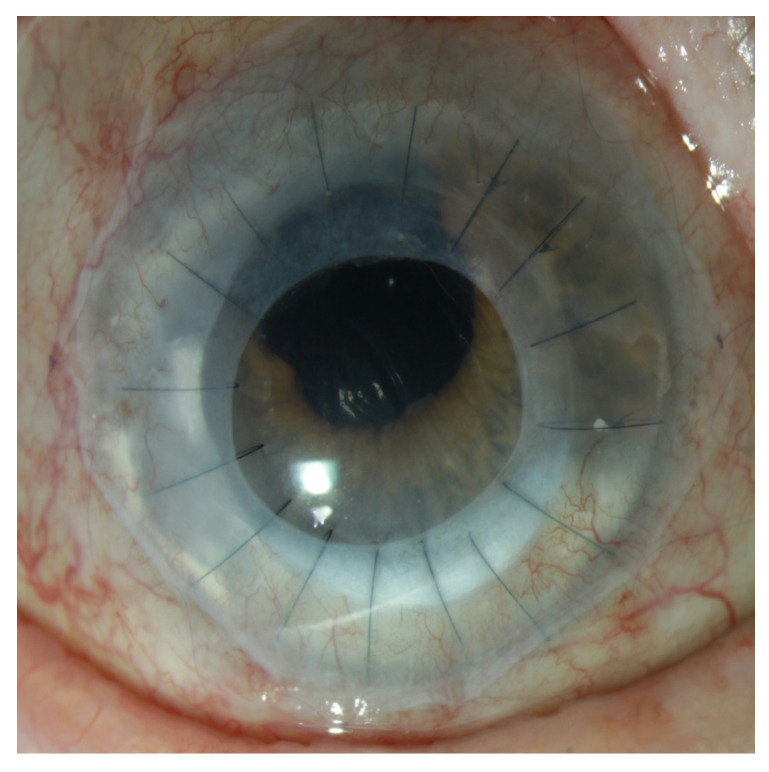
Schematic presentation of a penetrating limbo-keratoplasty and amniotic membrane transplantation. In this case, the limbal part of the transplant is oriented downwards, and the amniotic membrane is prepared with a central hole to improve vision, given the patient’s monocular situation.

All patients underwent limbo-PK by the same surgeon under general anesthesia and were hospitalized for two to three nights. Eccentric trepanation was performed on the donor corneoscleral button (Figure 1 and Figure 2). The graft was then fixed centrally with 16 to 32 single nylon 10.0 stitches. Finally, an amniotic membrane (AM) patch with a diameter of 15 mm was placed over the cornea and sutured using 4 single 10-0 Vicryl stitches and one running suture. A bandage contact lens (Ø 20.5 mm) was placed on the ocular surface for the following 4 weeks.

All patients received topical postoperative immunosuppressive therapy (0.66 mg unpreserved dexamethasone dihydrogen phosphate disodium, 6 times a day) along with antibiotic eye drops (ofloxacin) for at least 1 week and artificial tears at least six times per day. In ten of the fourteen cases, systemic immunosuppressive therapy with either prednisolone (1 mg per kg body weight tapered by 20 mg every 5 days) and/or mycophenolatmofetil (MMF) (1 g twice daily) was taken. The decision regarding the application and method of systemic immunosuppression was made by the surgeon based on the clinical risk assessment.

During the period of immunosuppression, patients underwent regular check-ups and blood tests by their general practitioner to promptly identify any potential side effects of the medication. Unless serious side effects occurred, immunosuppression with MMF was continued for 3–6 months.

The recipients were followed up at regular intervals in our outpatient clinic and underwent slit lamp examination to detect symptoms of immune reactions, graft rejection, graft failure, or infection. Furthermore, intraocular pressure and fundus were examined at each visit.

### 2.4. Statistics

Descriptive statistics were conducted using absolute and relative frequencies for categorical data and median and range and mean and standard deviation for continuous data. The difference between pre- and postoperative BCVA was evaluated by the Wilcoxon test.

## 3. Results

### 3.1. Patients’ Characteristics

We included 14 eyes of 13 patients aged 59.8 ± 14.1 years (range: 39 to 83 years), 4 females. During the evaluated four-year period, we performed approximately 350 PKs. Indications for LSCD included chemical burns (*n* = 4), blast injuries (*n* = 4), thermal burns (*n* = 2), trauma (*n* = 1), graft-versus-host disease (*n* = 1), postoperative LSCD (*n* = 1), and ectrodactyly-ectodermal dysplasia (EEC) (*n* = 1). Five of the 13 patients suffered from bilateral LSCD. Eleven of the fourteen eyes had undergone multiple surgical procedures, including cataract surgeries (*n* = 8), penetrating keratoplasties (*n* = 10, four of them HLA-typed in #5, #9, #10 and #14), allogeneic simple limbal epithelial transplantations (allo-SLET, *n* = 5), amniotic membrane transplantations (AMT, *n* = 7), and cauterization (*n* = 1), prior to limbo-PK. Five eyes had secondary glaucoma.

The mean preoperative BCVA was 2.2 ± 0.6 logMAR (range: light perception to 0.7 logMAR), in these patients, limbo-keratoplasty was performed. Mean size of the limbo-keratoplasty-graft was 8.79 ± 0.68 mm (range: 7.25–9.5 mm). All Limbo-PKs were combined with AMT; three with additional cataract surgery and one with tarsorrhaphy. Postoperatively, all eyes received local immunosuppression, and 10 patients received additional systemic immunosuppression with either mycophenolate mofetil (MMF) only (*n* = 5), systemic prednisolone only (*n* = 2), or both (*n* = 3).

### 3.2. Outcome

**Figure 3:**
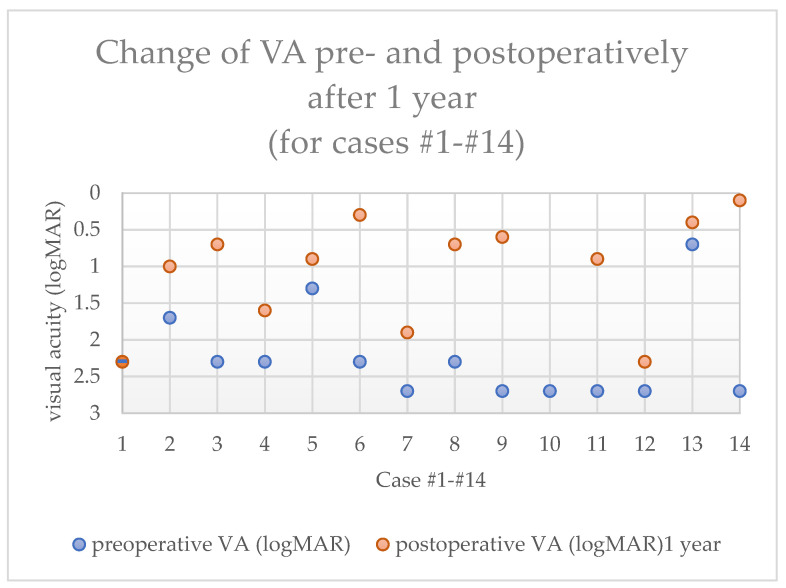
Visual acuity (VA) of each case numbered from 1 to 14 (cases one and two belonged to the same patient) measured pre- and postoperatively after one year. In case #1, visual acuity was the same preoperatively, and one year after Limbo-PK, in case number 10, no VA was recorded one year postoperatively.

Visual acuity in the first five postoperative days was poor (hand movement to counting fingers) as expected, since AMT was performed in all cases. After dissolution of the amniotic membrane after 3–4 weeks, a rapid increase in visual acuity was observed in most cases. After one year, mean postoperative BCVA increased significantly to 1.0 ± 0.7 logMAR (range: 2.3 to 0.1, *p* = 0.03) compared to preoperative BCVA (Figure 3). The mean follow-up time of the last consultation in this study was 29.4 months ±12.6 (range: 10 to 52 months), and the mean BCVA at the last consultation was 1.6 ± 0.8 logMAR (range: 2.7 to 0.4).

**Figure 4:**
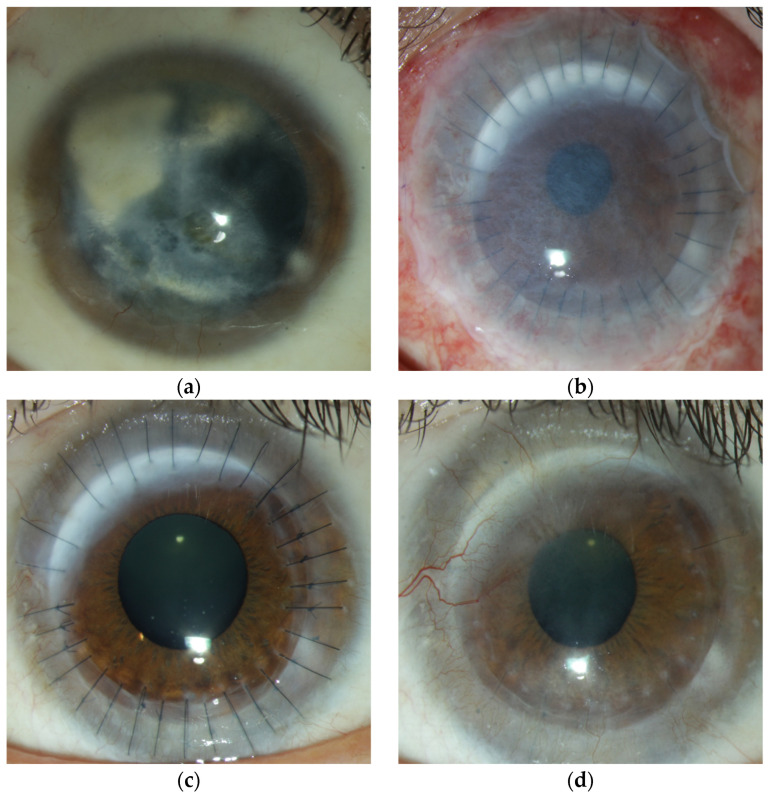
Eye number six received limbo-PK due to a chemical burn via mortar. (**a**): preoperative image of the anterior segment with corneal clouding and deep stromal opacities. (**b**): anterior segment one day after limbo-PK and AMT, 0.7 logMAR. (**c**): anterior segment with clear graft, limbal segment from 9 to 1° and tight sutures one year after limbo-PK, 0.3 logMAR. (**d**): 38 months of follow-up with vascularization of the graft from 9° and 11° reaching the center of the graft, with closed epithelium. He then received cauterization of the corneal vessels.

**Figure 5:**
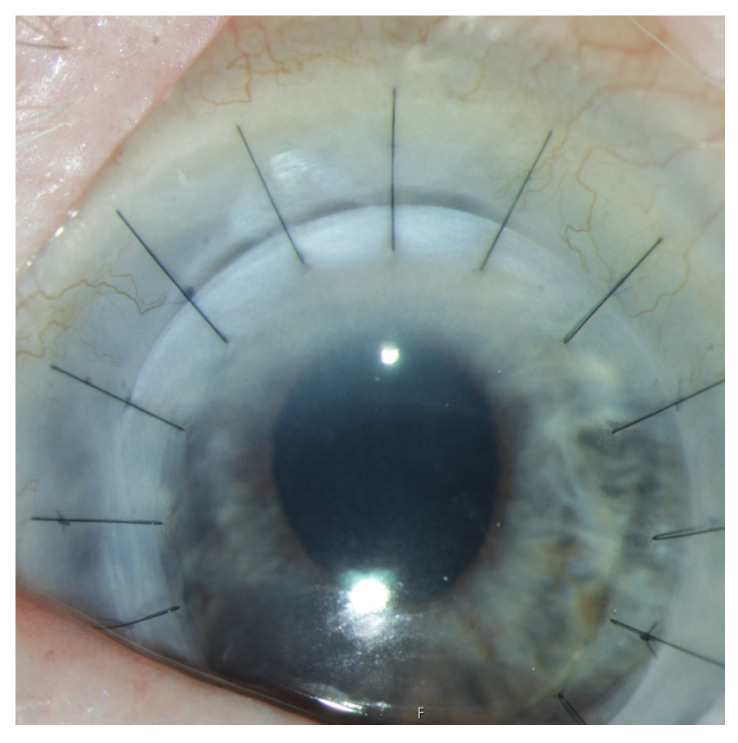
Eye number 13 received limbo-PK due to EEC syndrome. She had already undergone multiple surgeries in advance, including AMT, PK, and Allo-SLET. After Limbo-PK, she showed a clear graft with tight sutures and a closed epithelium one year postoperatively.

Eleven of the fourteen eyes showed a very satisfactory postoperative course. Especially patients without previous ophthalmologic surgery experienced an increase in visual acuity. The clinical course and treatment of eyes number 6 and 13 are shown in Figure 4 and Figure 5 as examples.

The re-epithelialization findings at the 1-year follow-up, and afterwards if longer follow-up was available, as well as the need for additional procedures, are listed in Table 1 for each patient. At 1-year follow-up, 11 of 14 (78.6%) grafts were clear with closed epithelium.

The clinical course of the other three eyes is described below:

In eye number 5, two PKs had taken place in advance, and two AMTs had been necessary in the postoperative course due to a healing disorder of the epithelium. At the last follow-up (after 42 months), graft failure was observed with persisting corneal erosion, vascularization, and opacification, requiring Re-Re-Re-Limbo-PK + AMT. Eye number 7 showed corneal erosion and a beginning graft failure, which could be prevented by local steroid therapy. At the last follow-up (after 22 months), it showed signs of graft immune rejection and corneal erosion, since the patient had stopped applying the local immunosuppressive therapy. We recommended a Re-Limbo-PK but did not perform the surgery yet due to the patient’s wish.

In the case of eye number 12, nine PKs had already taken place in advance due to a severe GvHD with recurring LSCD. In the postoperative course of the described limbo-PK, the eye showed a new corneal ulcer and vascularization due to persisting LSCD, requiring Re-PK à chaud (after 6 months) and Re-Limbo-PK (after one year).

**Figure 6:**
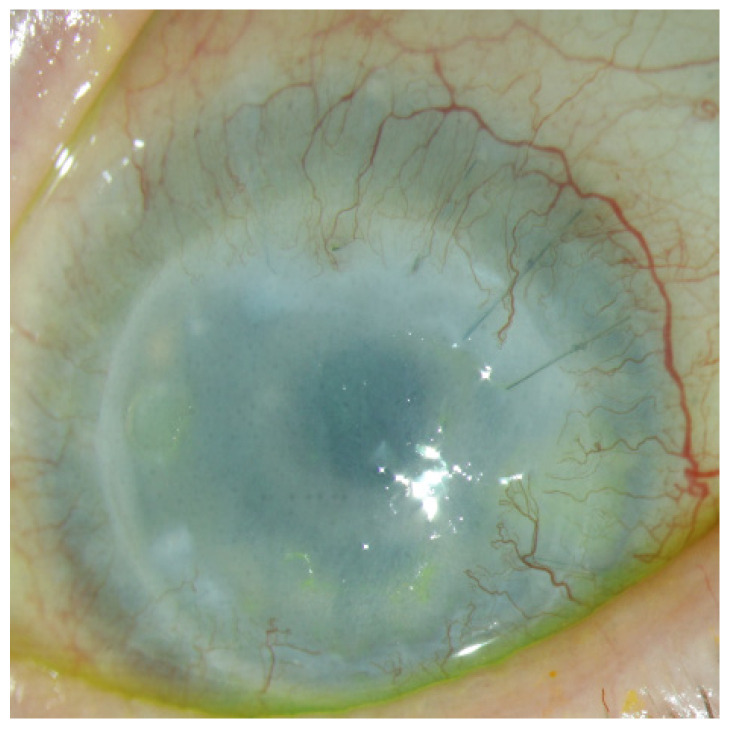
Eye number 14 presents a cloudy and vascularized transplant due to the recurrence of LSCD 39 months after Limbo-PK.

In total, at the last follow-up (10 to 52 months after PK), 9 of 14 eyes (64.3%) showed functioning grafts with closed epithelium. Five eyes showed epithelial defects due to graft failure: two eyes (number 7 and 10) due to graft immune rejection, and three (5, 12, and 14) due to recurring LSCD. In the two eyes (numbers 7 and 10), we suspected immune rejection with corneal edema and deterioration of the VA after limbo-PK. Both patients had ended immunosuppressive therapy early. In eye number 10, VA increased after reapplying immunosuppressive therapy, and no further surgeries were necessary. In eye number 7, graft rejection could not be prevented, and HLA-typed Re-Limbo-PK in combination with cataract surgery and AMT was recommended. Number 14 had already received four PKs in advance and showed a clear graft and satisfactory VA (0.1 logMAR) until the patient stopped the ophthalmologic check-ups in our clinic and presented again after 39 months with a cloudy and vascularized transplant due to recurrence of LSCD (Figure 6).

**Figure 7:**
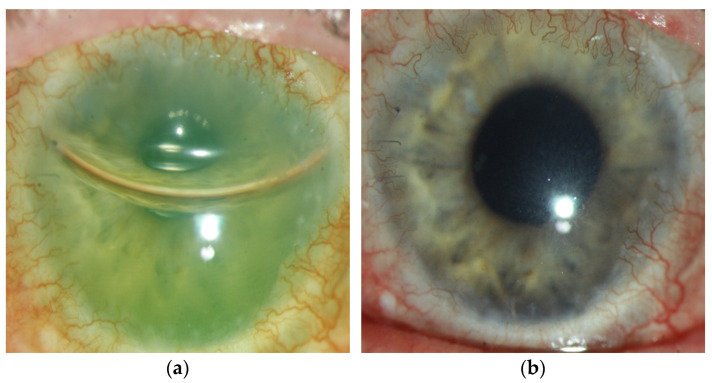
Eye number 2 showed endothelial decompensation, requiring a DMEK. (**a**) postoperative image of the anterior segment with corneal clouding and a gas fill of 50% in the anterior chamber. (**b**) anterior segment three months after DMEK, showing a clear cornea after limbo-PK and DMEK.

Eye number 2 showed a closed epithelium, but graft failure with corneal edema due to endothelial decompensation, requiring a DMEK. He showed a clear graft after DMEK (Figure 7).

## 4. Discussion

The aim of our study was to report on and to evaluate our clinical practice with limbo-PK. Compared to older methods of limbo-PK, where oversized grafts with circular limbal stem cells were usually implanted, the newer, central limbo-PK we performed is less traumatic [14]. Due to the smaller diameter of the transplant and only partial transplantation of stem cells, fewer immune reactions are provoked, and satisfactory postoperative results can be achieved [15]. The mean size of the corneal graft was 8.8 mm in our study, which is well comparable to the study of Reinhard et al., who initially used this new technique in 1996. In their study of 1999, they described their grafts with diameters between 7.7 and 10.0 mm [8].

Nevertheless, postoperative outcome varies greatly between different publications that have been issued since then. These independent nonrandomized case series showed heterogeneous compositions of the patient population regarding the extent of stem cell damage, drug therapy, genetic background, time of surgery, surgical method, and follow-up.

When comparing the results of our (also heterogeneous) cohort to those described in the literature, we were able to achieve satisfactory postoperative results after central limbo-PK with a closed epithelium in 79% of eyes after one year. After a mean follow-up of 27.4 months, 64.3% of eyes showed a still-functioning graft. In a cohort study with 206 eyes, Lang et al. report outcomes after Limbo-PK comparable to ours. After 2.5 years, a clear graft could be detected in 80% of cases after limbo-PK due to LSCD [16]. What is outstanding about this study is the long follow-up period: After 19,1 years, no clear graft was documented in the patient group that received Limbo-PK due to LSCD. Patients with corneal dystrophies showed clear graft survival with 90% after 5 years [16].

In contrast to this satisfactory outcome after 2.5 years, Reinhard et al. found postoperative graft failure in 18 of 25 eyes in their monocentric cohort study from 2004 after homologous penetrating central limbo-keratoplasty (HPCLK) [8]. In this study, too, the limbo-PK took place due to LSCD, and the follow-up period of 12–41 months was comparable to that of our study, which ranged from 10 to 52 months. Although all patients in the study of Reinhard et al. received systemic immunosuppression with cyclosporin A (CSA) for at least one year postoperatively, immune rejection and resulting surface disorders could not be prevented. While the short-term results of the study were very promising and the majority of transplants were clear after 9.6 months, the high rate of graft failure became apparent at the end of the follow-up (after 2 months at the earliest and 29 months at the latest) [8]. Our study showed very good postoperative results after one year, but during further follow-up, the number of cases with epithelial healing disorders with recurrence of LSCD or graft rejections rose to 5 of 14 eyes after a period of 29.4 ± 12.7 months. This development suggests that, over time, the reservoir of transplanted limbal stem cells decreases and is not able to generate sufficient epithelial cells, leading to new epithelial defects. We observed graft rejection only in two patients who discontinued immunosuppressive therapy.

From an immunological point of view, the cornea occupies a kind of special position within transplantation medicine. Its risk of rejection is often described as comparatively low compared to that of other organs. This is due to the avascularity of the cornea. Absence of vascular and lymphatic vessels limits contact with the recipient’s immune system [17]. Furthermore, the anterior chamber fluid fulfills an immunosuppressive function: Anterior Chamber Associated Immune Deviation (ACAID) suppresses cellular components of a type IV immune reaction (late-type immune reaction) against the transplanted tissue [18].

Postoperatively, the transplants are endangered by immune reactions against both the endothelial cells and the limbal stem cells [8]. Immune rejection continues to be the primary cause of graft failure after “conventional” PK (without the limbus), responsible for more than half of graft loss cases. The survival rate in inflamed, vascularized host beds, so-called high-risk (HR) corneal transplantation—including Limbo-PK—decreases to below 35%.

Therefore, it seems logical that in cases where LSCs are transplanted due to LSCD, the recipient’s immune response to the donor corneal stem cells should be suppressed. Lifelong topical and even systemic immunosuppressants may be administered prophylactically to inhibit or regress corneal lymphangiogenesis and hemangiogenesis and to prevent immune-mediated graft rejection, if no undesirable side effects occur [19,20].

In the study of Reinhard et al., systemic treatment with 60–100 mg of fluocortolone was administered for three weeks postoperatively and given in the postoperative course if an immune reaction against a graft was suspected. CSA was administered to every patient for at least one year. Topical corticosteroids (prednisolone-21-acetate 1% eye drops) were administered in doses of five to seven drops daily, three drops over the long term. They observed 18 graft failures in their study of 25 patients. A total of 15 of these patients were still receiving systemic CSA at the time of graft failure. Reasons for the 18 graft failures included severe surface disorders, endothelial immune reactions, and a combination of surface disorders and endothelial immune reactions [9].

The authors of this publication suspected that the frequency of graft failure was related to the potency of the immunosuppressive postoperative medication and to the therapy adherence of the patients. This thesis is supported by our findings: We administered more potent systemic immunosuppressants (either mycophenolate mofetil, Decortin H, or both) for three to six months to our patients postoperatively and achieved significantly lower postoperative rejection rates. It is therefore even more important to provide patients with consistent education about the necessity of local immunosuppressive therapy. Contrary to the authors’ belief that postoperative systematic immunosuppression is only justified in high-risk patients after limbo KPL, not only our study, but also common literature shows that a lower postoperative rejection rate can be achieved also for non-high-risk patients with long-term systematic immunosuppression. Birnbaum et al. showed a statistically significant, stronger effect of MMF compared with CSA in preventing immune reactions after high-risk keratoplasty [21]. Furthermore, our study confirmed Decortin H as a sufficient systemic immunosuppressive single therapy for improving transplant survival: Four of our 14 patients were prescribed Decortin H only, and three of these patients showed a satisfactory postoperative outcome with closed epithelium.

A combination of limbo-PK with mitomycin C, amniotic membrane, and conjunctivoplasty is suggested by Eberwein et al. in their report on 20 cases with bilateral LSCD [15]. They were able to achieve overall clear grafts and graft survival in 14 out of 20 cases. Thus, the surgical success rate of their study was 70%. Measured by the rate of necessary reoperations after limbo PK in our study, only four cases showed either epithelial healing disorder, requiring repeated amniotic membrane suturing, endothelial decompensation requiring DMEK, or vascularization of the cornea requiring cauterization of vessels. Two patients experienced a graft failure, whereas one of the patients did not apply the topical corticosteroids as prescribed. One patient did not take the systemic steroid therapy as prescribed and showed graft immune rejection after six months. We achieved these results with a combination of limbo-PK with amniotic membrane transplantation for every patient and achieved better surgical results compared to Reinhard et al., who performed simple limbo-PK without AMT in most cases.

It is also important to note that Reinhard et al. did not perform any HLA-typed limbo-PK in their study, as this work reviewed the early results of this surgical procedure. While rejection prophylaxis through matching for human leukocyte antigens (HLA) is controversial in “isolated” PK, meanwhile, it has been shown to improve graft survival in limbo-PK, especially in specific, high-risk cases [12]. However, the low availability of corneal transplants, in general, as well as the additional waiting time and low HLA matchability are reasons for the rare use of HLA-matched transplants, in general [22]. Four of our 14 cases received HLA-typed grafts. Three of these cases were chosen for HLA typing because they had already experienced graft failure in the past. Although the patients who received HLA-typed transplants were considered “high-risk keratoplasty”, two of the four eyes (eyes 9 and 10) showed a very satisfactory postoperative course with closed epithelium after one year and at the time of the last follow-up (12 and 35 months). They did not require reoperation and showed a clear transplant and a satisfactory postoperative course. However, eyes numbered 5 and 14 required reoperations due to graft failure and persisting corneal erosion and corneal ulcer.

Ten of the fourteen eyes in our study achieved a VA of at least 1.0 logMAR one year postoperatively. At the time of the last follow-up, five of the 14 eyes achieved a VA of at least 1.0 logMAR. Lang et al. found a vision better than 20/200 ft (equivalent to 1.0 logMAR) in 50% of their 150 patients after 3–7 years, which is comparable to the results after one year in our study. Lang et al. found rejection-free graft survival of 43% for eye burns, 25% for congenital aniridia, 30% for inflammatory disease, and 68% for all other indications [12]. We suspected graft rejection in one case (eye number 7), which received Limbo-PK due to a thermal burn. However, in this case, we do not attribute the poor postoperative course to the pre-diagnosis, but to premature discontinuation of steroid therapy. Other grafts in the study remained clear for more than four years, which correlates with the prescribed median graft survival in the literature of 3 to 4 years [12,16].

We observed a relationship between preoperative factors and poor postoperative graft survival: Patients with previous PK in their medical history appeared to have a higher risk of graft failure. In our study, more than half of the eyes that had already undergone at least one PK prior to surgery showed either graft failure or a lack of epithelial closure after Limbo-PK. Furthermore, the diagnosis of GvHD seemed to be a high-risk factor for postoperative graft failure. The patient suffering from severe GvHD had already experienced eight PKs in his past and showed a particularly poor postoperative outcome with a renewed perforation after his Limbo-PK. In cases of GvHD or multiple ophthalmological operations in the medical history, HLA-typed limbo-PK or keratoprosthesis should therefore be considered. In the case of eye number 12, HLA-typed limbo-PK could not be waited for due to a perforation of the graft. However, most operations were performed due to LSCD following trauma, blast injuries, or thermal/chemical burns. For these cases, no correlation with the postoperative outcome could be found in the cohort.

## 5. Limitation

Limitations of our study lie in its retrospective design and the limited number of patients. Limbo-PK is a surgical procedure applied to only a small number of patients who have already undergone multiple unsuccessful therapies. During the four-year study period, Limbo-PK accounted for only about 4% of all penetrating keratoplasties performed in our clinic, underscoring the rarity of this indication. The small study cohort, despite the long observation period, does not allow for a robust statistical analysis. Furthermore, a control group of PK was not tested for postoperative results due to the poor comparability: Limbo-PK was reserved for a specific and therefore small group of patients suffering from LSCD who had already undergone previous corneal surgeries like PK or allo-SLET and had shown unsatisfactory results (e.g., recurrence of LSCD). Therefore, they differed greatly from the patient group for whom PK was indicated.

As VA is dependent on several factors, other vision-related diseases must be included in the analysis. The high rate of preoperative glaucoma (46%) is noteworthy in the evaluation of the postoperative visual acuity, with four of the five patients already having a history of glaucoma surgery (three patients underwent controlled cyclophotocoagulation and one underwent filtering surgery). The postoperative potential of visual acuity after limbo-PK was therefore not only influenced by a clear cornea and a functioning graft but also by the extent of optic nerve damage.

## 6. Conclusions

In conclusion, limbo-PK is an effective vision-restoring surgical treatment of LSCD. It may be considered as an alternative or as an interim solution prior to deciding on a keratoprosthesis. The survival of the graft relies on the viability of the transplanted limbal stem cells. To enhance graft longevity, local and systemic immunosuppressive therapy should be employed. In cases that had undergone previous keratoplasties, HLA typing should be considered. We were able to achieve a significant improvement in the mean BCVA of the patients, and nearly all of them (79%) showed a closed epithelium after a one-year follow-up.

Nevertheless, limbo-PK is a surgical procedure that is highly invasive and carries risks such as graft failure or immune rejection, infections, or secondary glaucoma. It should therefore be reserved for patients with total limbal stem cell failure, for whom less invasive techniques such as limbal stem cell transplantation or amniotic membrane suturing will not result in a better VA.

## Figures and Tables

**Table 1:** Characteristics and postoperative outcome of patients who received penetrating limbo-keratoplasty (limbo-PK).

Eye	Diagnosis	Graft Size [mm]	Previous Procedures	Preoperative BCVA	Surgery	Post-Operative VA at T1 (Days)	BCVA at 6 Months Follow Up	BCVA at 12 Months Follow Up	BCVA at Last Follow Up (Months)	Epithelium 1 Year Postoperatively	Epithelium at Last Follow-Up	Additional Procedures Following Limbo-PK and VA-Relevant Comorbidities
#1	blast injury	9.5	HLA-typed PK, alloSLET, AMT, CoCo, cataract surgery	2.3	Limbo-PK + AMT + IOL-Explantation	1.2 (30)	2.3 (6)	2.3 (12)	2.3 (26)	closed	closed	CoCo + cryocoagulation due to secondary glaucoma
#2	blast injury	9.2	HLA-PK, alloSLET, DMEK, CoCo, AMT, cataract surgery	1.7	Limbo-PK + AMT	0.5 (30)	1.6 (4)	1.0 (12)	1.6 (52)	closed	closed	DMEK, CoCo + cryocoagulation due to secondary glaucoma
#3	chemical burn	7.25	EDTA-Abrasion x4, Trabeculectomy, cataract surgery	2.3	Limbo-PK + AMT	1.4	0.2 (6)	0.7 (12)	0.5 (43)	closed	closed	CoCo + cryocoagulation
#4	thermal burn	9.0	2x PK, AMT, cataract surgery	2.3	Limbo-PK + AMT	1 (45)	1 (6)	1.6 (12)	2.3 (17)	closed	closed	-
#5	blast injury	9.0	2x PK, AMT	1.3	Limbo-PK + AMT	0.7 (16)	0.8 (6)	0.9 (12)	2.7 (42)	corneal erosion requiring AMT	corneal erosion	Re-Limbo-PK + AMT (planned) due to graft failure (persisting corneal erosion + vascularization + opacification
#6	chemical burn	8.75	-	2.3	Limbo-PK + AMT	0.5 (90)	0.6 (5)	0.3 (12)	1,3 (38)	closed	closed, (vascularisation)	Cauterisation of vessels
#7	thermal burn	9.2	PK, EDTA-Abrasion	2.7	Limbo-PK + AMT	2.3 (90)	1.9 (6)	1.9 (12)	1.9 (22)	corneal erosion and beginning graft failure (improvement through local therapy)	corneal erosion, graft immune rejection	Re-Limbo-PK recommended after immune rejection due to missing local immunosuppressive therapy (not yet due to patient’s wish)
#8	blast injury	9.5	-	2.3	Limbo-PK + AMT + cataract surgery	0.7 (30)	0.7 (6)	0.7 (14)	0.7 (13)	closed	closed	-
#9	chemical burn	8.25	-	2.7	Limbo-PK + AMT + cataract surgery	1,6 (30)	0.5 (6)	0.6 (12)	0.6 (12)	closed	closed	-
#10	trauma	9.5	3x PK, cauterisation of vessels, alloSLET, CoCo, Cataract surgery	2.7	Limbo-PK + AMT		2.3 (6)	2.3 (14)	2.3 (14)	closed	closed	-
#11	chemical burn	8.25	PK, AMT, alloSLET, Cataract surgery	2.7	Limbo-PK + AMT	1.3 (30)	1.3 (6)	0.9 (11)	1.5 (35)	closed (vascularisation)	corneal erosion (treated with Serum eyedrops)	Cauterisation of vessels
#12	GvHd	9.5	9xPK, AMT, CoCo, cataract surgery	2.7	Limbo-PK + AMT + tarsorrhaphy	2.3 (30)	2.3 (4)	2.3 (10)	2.7 (10)	corneal ulcer, severe GvHD and LSCD	corneal ulcer, severe GvHD and LSCD	Re-PK (after 6 months) and Re-Limbo-PK (after one year) after perforation of the graft due to severe GvHD and LSCD
#13	EEC-Syndrome	7.75	AMT, PK, alloSLET, cataract surgery	0.7	Limbo-PK + AMT	0.7 (30)	0.5 (6)	0.4 (12)	0.4 (27)	closed	closed	--
#14	postoperative LSCD	8.5	4x PK (last one HLA-typed)	2.7	Limbo-PK + AMT + Cataract surgery	0.8 (30)		0.1 (12)	2.3 (39)	closed	Epithelial defects, recurrence of LSCD	Re-Limbo-PK is planned for 2025 due to graft failure (cloudy and vascularised transplant, recurrence of LSCD
Mean		8.79 ± 0.68		2.17 ± 0.6		1.15	1.23	1.05 ± 0.7 (*p* = 0.03)	1.61 ± 0.8 (27.4)			

Pre- and postoperative best-corrected visual acuity (BCVA) (logMAR) after limbo-PK at times 1 (T1) to 3 (T3). PK—penetrating keratoplasty, AMT—amniotic membrane transplantation, GvHD—graft versus host disease, CoCo—controlled cyclophotocoagulation, mm—millimeters, m—months, d—days.

## Data Availability

Data are available on request.

## Abbreviations

The following abbreviations are used in this manuscript:

Lsc	Limbal Stem Cell
Lsd	Limbal Stem Cell Deficiency
Acaid	Anterior Chamber Associated Immune Deviation
Am	Amniotic Membrane
Bcva	Best Corrected Visual Acuity
Va	Visual Acuity
Limbo-PK	Limbokeratoplasty
Pk	Perforating Keratoplasty
Csa	Cyclosporin A
Hpclk	Central Limbo-Keratoplasty
Allo-SLET	Allogeneic Simple Limbal Epithelial Transplantation
Amt	Amniotic Membrane Transplantation
Coco	Controlled Cyclophotocoagulation
Dmek	Descemet Membrane Endothelial Keratoplasty
Te	Trabeculectomy
TGFBI-Gene	Transforming Growth Factor Beta-Induced Gene
Gvhd	Graft-Versus-Host Disease
